# Neighborhood Access to the Built Environment and Allostatic Load: A Systematic Review of the Use of Geographic Information Systems

**DOI:** 10.3389/phrs.2024.1606624

**Published:** 2024-05-23

**Authors:** Owen Cranshaw, Steven Haworth

**Affiliations:** Institute for Social and Economic Research (ISER), University of Essex, Colchester, United Kingdom

**Keywords:** allostatic load, geographic information systems, built environment, biomarker, GIS

## Abstract

**Objectives:** This paper systematically reviews how spatial analysis has been used to measure relationships between access to the built environment and Allostatic Load (AL) or biomarkers relevant to the stress pathway. Geographic Information Systems (GIS) facilitate objective measurement of built environment access that may explain unequal health outcomes linked to living in stressful environments.

**Methods:** Systematic review, search date 13 July 2022 with methods published *a priori*. Included studies that quantitatively assessed associations between GIS measures of neighborhood attributes and biomarkers of stress.

**Results:** 23 studies from 14 countries were included having used GIS measures to assess relationships between access to the built environment and biomarkers relevant to AL, with 17 being cross-sectional and 6 longitudinal. Just 2 studies explicitly assessed associations between GIS measures and AL, but 21 explored biomarkers relevant to the stress pathway. GIS was used to calculate density (how much of x within y) and proximity (how far from a to b) measures.

**Conclusion:** GIS measures of greenspace, the food environment, area-level demographics, and land-use measures were found to influence biomarkers relevant to the stress pathway, highlighting the utility of this approach. GIS use is extremely limited when measuring the built environment and its influence on AL but has been widely used to consider effects on individual biomarkers of stress.

**Systematic Review Registration:** [https://www.crd.york.ac.uk/prospero/display_record.php?RecordID=348355], identifier [CRD42022348355].

## Introduction

A growing literature indicates increasing geographical inequalities in health, with the worst outcomes occurring for those born and living in the most deprived neighborhoods [1]. For example, the 2010 Marmot Review found that, on average, those living in the most deprived neighborhoods die 7 years younger than those living in the least deprived neighborhoods. Moreover, the Marmot 10 years follow up found inequalities in life expectancy had increased, particularly in the most deprived neighborhoods [2]. The effects of neighborhood characteristics on various health outcomes have been shown to persist even after adjusting for multiple individual factors such as age, gender, ethnicity and educational attainment [3–10]. As a result, where we live is increasingly being recognized as one of the most important precursors of chronic disease, and as an explanation for variations in health outcomes [11].

Yet, most neighborhood effects studies fail to define the causal mechanisms that explain how neighborhood characteristics contribute to increasing risks of poor health over and above individual risk factors [12, 13]*.* For instance, poorer quality housing may explain risk at an individual level, but how a neighborhood having a higher percentage of low-quality housing contributes to more widely distributed poorer health outcomes across the area (even to those not exposed directly) is less well understood. Moreover, studies often include only a limited range of neighborhood characteristics, masking complexity and hindering their ability to identify neighborhood-level determinants of health outcomes [14]*.* Responding to such critiques, studies have sought to identify potential causal pathways, with growing evidence pointing to the stress pathway, theoretically measured by the concept of Allostatic Load (AL), playing a mediatory role between neighborhoods and poorer health outcomes [1, 15, 16]. The stress pathway encapsulates the idea that adverse neighborhood environments, which may be characterized by social or economic disadvantage, neighborhood disorder, or fewer or poorer quality resources, can act as chronic stressors that activate and lead to dysregulation of the hypothalamic-pituitary-adrenal-axis [17]. Furthermore, AL is conceptualized as the overall wear and tear on the body, resulting from stressors in the environment and as a bridge to poorer health outcomes. As such, where environmental stressors are more pronounced or ongoing, the stress response is likely to be activated more frequently, increasing the likelihood of dysregulation across a range of markers of the stress response.

Advances in Geographic Information Systems (GIS), and recognition of the utility of the approach, make it possible to consider which attributes of neighborhoods might contribute to poorer health outcomes by objectively and accurately measuring the distribution of, and access to, specific resources within neighborhoods. GIS techniques provide the potential to capture the complexity of community environments at finer scales than existing measures of deprivation, offering the potential to identify target areas for policy interventions. As such, there is an increasing body of evidence using GIS to consider how where we live impacts health [18–20]. One complexity is that GIS measures are wide-ranging, since they can incorporate any data that has a spatial attribute. Therefore, GIS has been applied in a variety of ways, highlighting the need for this review.

This review brings together these strands of neighborhood effects research and reviews the evidence of how GIS has been used to measure relationships between access to the built environment and biomarkers associated with stress or overall AL. Given the increasing body of evidence pointing to the stress pathway linking neighborhood effects on health, and the benefits of using GIS to measure the complex structure of neighborhoods, this review seeks to elucidate how spatial analysis has and can be used and its effectiveness when exploring the relationship between place and health.

## Methods

This systematic review conforms to the Preferred Reporting Items for Systematic Reviews and Meta-analyses (PRISMA) Guidelines [21] and was registered with PROSPERO with protocol registration number: CRD42022348355.

### Search Strategy

Search terms were clustered under three overarching themes: “Geographic Information Systems,” “Neighborhood Effects” and “Allostatic Load or Stress.” Search terms were informed by relevant systematic reviews [11, 16, 20, 22, 23]. All, included studies were required to be empirical; human; available in English; and to quantitatively assess associations between GIS measures of neighborhood attributes and biomarkers of stress or overall AL. Terms like “GPS” and “Accelerometer” measures were not included in the search terms as these were considered to capture individual level, rather than neighborhood level attributes of the built environment. Reviews and studies that did not contain explicit GIS measures or at least one stress or AL-related biomarker were excluded, as were studies focused solely on BMI. A detailed description of the search strategy and terms is available in the registered PROSPERO protocol [24]. Records were screened by both researchers independently and in duplicate, with near-perfect agreement (94%, Cohen’s k = 0.89) and discrepancies checked by an independent researcher.

### Data Sources

PubMed; MEDLINE; PsychInfo; PsychArticles; CINAHL; Scopus; Web of Science, and the pre-print journal databases; ESSOAR and medRxiv were searched from database establishment to 13th July 2022. Database searching was accompanied by grey literature searches and hand searching of reference lists of included studies. Grey literature included searches through working papers from research institutes, and reports, briefs, and policy documents from relevant agencies [e.g., Office for National Statistics (ONS)].

### Risk of Bias Assessment and Data Extraction

Data extraction was completed independently and in duplicate by Author-1 and Author-2. A predetermined and standardized Excel spreadsheet was used to extract: author, publication date, and title; country; population characteristics and sample size; study design; data sources for population and neighborhood data; neighborhood unit; outcome measure/s; GIS measures, results (including direction and strength); and study strengths and limitations. Extraction records were subsequently combined, with discrepancies discussed and agreed upon. Risk of bias for all included records was assessed independently and in duplicate by both researchers using the Joanna Briggs Institute (JBI) appraisal tools [25]. Records were rated as having a low, moderate or high risk of bias with discrepancies checked by an independent third researcher.

### Analysis

The focus of this review is to identify how GIS methods have been used in assessing relationships between neighborhood characteristics and biomarkers relevant to AL. Therefore, methodological variations used in this field were thematically analyzed. Results were organized by the type of GIS measure used (density, proximity), and subcategorized by the aspect of the neighborhood context these measures sought to capture.

## Results

### Characteristics of Selected Studies

**FIGURE 1:**
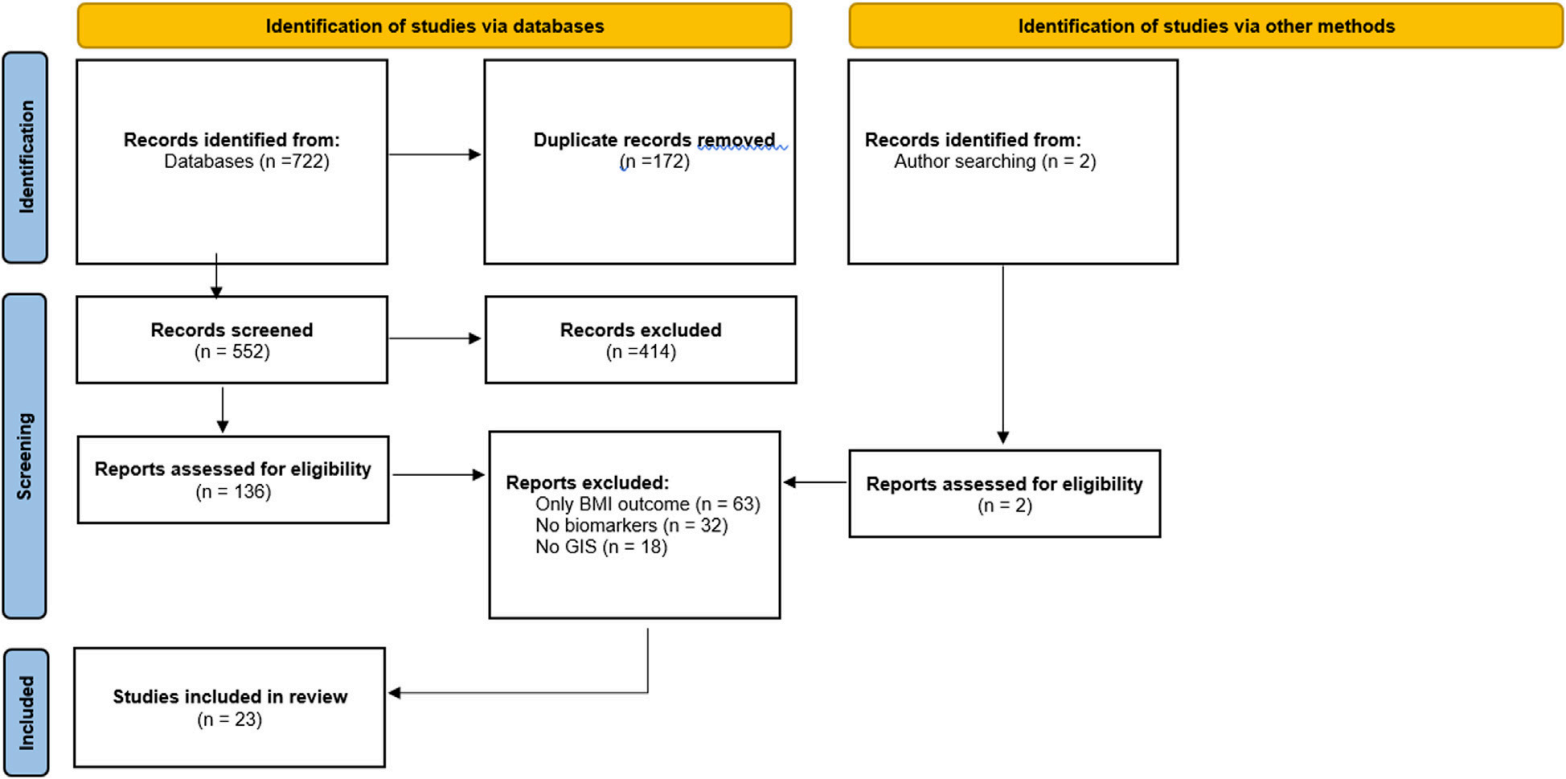
Flow diagram of inclusion/exclusion (systematic review, worldwide, journal start-date-2022).

722 records were identified through database searching with 2 found through reference list searching. Of these 724 records, 172 duplicates were removed, leaving 552 unique records. Title and abstract screening further removed 414 records. The remaining 138 studies were obtained for full-text screening. During full-text screening, 63 records were excluded for only having BMI as an outcome, 32 for having no biomarkers, and 18 because they had no explicit GIS measures. The remaining 23 articles were included for review (see Figure 1).

The 23 included studies were conducted in 14 countries including: Australia [26], Canada [27–30], France [31], Greece [32], Japan [33], Portugal [34], South Africa [35], the United Kingdom [36], and the United States (US) [37–48]. Thirteen studies [26, 28, 29, 31, 32, 34–36, 38, 42–44, 48] were from 2017 or later. Five studies were longitudinal [36, 40, 43, 46, 47] and 18 were cross-sectional [26–35, 37–39, 41, 42, 44, 45, 48]. Twenty studies included adults only (at least 18+) [26–33, 35–40, 42–45, 47, 48], 2 included only children [34, 46], and 1 both [41]. Table 1 summarises the included studies.

**TABLE 1:** Study characteristics (systematic review, worldwide, journal start-date-2022).

Reference, country	Study design, sample and area	Data sources	GIS measure(s)	Outcomes(s)	Risk of bias
Baldock et al. [26] (Australia)	Design: Cross-sectional Sample: 1,491 adults, aged 18+ from North West Adelaide Area: Buffer: Residential Address, State Suburb	Population: • North West Adelaide Health Study (NWAHS) Neighborhood: • 2007 South Australian Retail Database • 2007 South Australian Property Cadastre • 2006 Australian Bureau of Statistics Census of Population and Housing	• Distance to: 1. Public Open Space 2. Food/vegetable Retailers • Discordance between perceived and objective distances to: 1. Food and vegetable Retailers 2. Public Open Space • Median household income	Metabolic Syndrome: • Waist Circumference • Triglycerides • HDLC • Dyslipidaemia, • SBP • DBP • Hypertension • Fasting glucose	Low
Naimi et al. [27] (Canada)	Design: Cross-sectional Sample: 342 adults, aged 18–55 from Montreal Area: Buffer: Residential Address, Census Tract	Population: • Montreal Neighborhood Survey of Lifestyle and Health Neighborhood: • 2001 Canadian Census data	• Area-level unemployment • Area-level education	Cardiovascular Risk: • HbA1c • Triglycerides • Total cholesterol • HDLC	Moderate
Hajna et al. [28] (Canada)	Design: Cross-sectional Sample: 2,809 Canadian adults, with a median age of 41.5 (SD = 15.1) Area: Buffer: Residential Address	Population: • Canadian Health Measures Survey (CHMS) Neighborhood: • Statistic Canada’s Postal Code Conversion File Plus (PCCF+, 2009 DMTI CanMap^®^ Streetfiles (DMTI Spatial Inc., Markham, ON, Canada) • 2006 Canadian Census Population Counts File	• Land-use Mix • Street Connectivity • Population Density • Neighborhood active-living environment index	Cardiovascular Risk: • BMI • SBP • Total cholesterol/HDL cholesterol ratio • HbA1c	Low
Walker et al. [29] (Canada)	Design: Cross-sectional Sample: 5,125 Canadian adults, aged 35+ with an IQR of 46–61 Area: Buffer: Residential Address, Dissemination Area	Population: • Prospective Urban Rural Epidemiology (PURE) Neighborhood: • 2006 Statistics Canada Census Data • United States Geological Survey’s EarthExplorer platform • OpenStreetMap	• Urbanity level • NDVI • Walkability Index • Density of Greenspace • Land coverage by green urban spaces (%) • Greyspace • DRI-GLUCoSE index • Area-level education • Area Level Lone Parent Families • Median Household Income • % Population in poverty • Area-level unemployment • Average Individual Income • % Dwelling Ownership • % Dwellings Rented • Labour force participation rate	• Fasting Blood Glucose	Moderate
Paquet et al. [30] (Canada)	Design: Cross-sectional Sample: 344 adults, aged 18–57 from Montreal Area: Buffer: Residential Address, Census Tract	Population: • Montreal Neighborhood Survey of Lifestyle and Health Neighborhood: • 2001 Canadian Census Data • 2003 Inventory of businesses and services	• Median Household Income • Area-level education • % Population in poverty • Density of Fast Food outlets	Metabolic Risk: • HDL • Total cholesterol • Waist Circumference • BMI • Triglycerides • HbA1c	High
de Courrèges [31] (France)	Design: Cross-sectional Sample: 3,218 French adults, aged 40–65 Area: Buffer: Residential Address	Population: • ELISABET survey Neighborhood: • 2010 French INSEE population census data • 2013 road data from the French National Institute for Geographic and Forest Information	• Urban Area • Walkability Index • Residential density • Street Connectivity • Land-use Mix • Distance to dining/drinking facilities • The WS (a proprietary walking index originally developed in the US) • Distance to: 1. Grocery Stores 2. Other shops 3. Places for errands 4. Parks 5. Schools 6. Culture/Entertainment • Population density • Block Length • Intersection density • Annual mean residential PM10 concentration • Median Household Income	Cardiovascular Risk: • BMI • Blood Pressure • Hypertension • HDLC • Cholesterol Level • LDLC • Triglycerides • SBP • DBP • HbA1c	Low
Tsiampalis et al. [32] (Greece)	Design: Cross-sectional Sample: 2,749 Greek adults, aged 18–89 Area: City Neighborhoods, Municipalities, Metropolitan Athens, Sector by SES	Population: • ATTICA epidemiological study Neighborhood: • 2016 Urban Atlas	• Case rate • Average Individual Income • Area-level unemployment • % of Illiterate population • % not completed primary school • Proportion of Immigrants (%) • Proportion of women (%) • Population mean age • Proportion married (%) • Area Socioeconomic Status Number of street markets • Number of supermarkets Green urban space land coverage (%) • Sports facility land coverage (%)	Metabolic Syndrome: • Waist Circumference • Triglycerides • HDLC • Cholesterol Level • Blood Pressure • Fasting glucose	Moderate
Hamano et al. [33] (Japan)	Design: Cross-sectional Sample: 1,348 adults from Okinoshima, with a mean age of 65 (SD = 6.9) Area: Network Buffer: Residential Address, population centre	Population: • Shimane COHRE Study Neighborhood: • Shimane COHRE Study	• Road Network Distance	Hypertension: • SBP • DBP	High
Ribeiro et al. [34] (Portugal)	Design: Cross-sectional Sample: 3,108 adults, with a mean age of 85 (SD = 2.3) Area: Buffer: Residential Address, Buffer: School	Population: • Generation XXI Neighborhood: • ArcGIS Online World Geocoding Service	• Accessibility • Residential area • Greenspace • Garden • Population density • Urbanity level • Density of greenspace Distance to greenspace	Allostatic Load: • CRP • HDLC • Total cholesterol • HbA1c • Waist-hip ratio • SBP • DBP	Low
Malambo et al. [35] (South Africa)	Design: Cross-sectional Sample: 341 adults, aged 35–70 from Cape Town Area: Buffer: Residential Address	Population: • Prospective Urban Rural Epidemiology (PURE) Neighborhood: • Data from the University of Cape Town Faculty of Engineering and Built Environment	• Distance to: 1. Community Centre 2. Transit Stops 3. Retail Shopping Centre 4. Public Open Space	Cardiovascular Risk: • BMI • DBP • SBP	Moderate
Sarkar et al. [36] (UK)	Design: Longitudinal Sample: 429,334 UK adults, aged 37–73 Area: Buffer: Residential Address, Buffer: Post Codes	Population: • UK Biobank Neighborhood: • UK Biobank Urban Morphometric Platform (UK BUMP) • Ordnance Survey AddressBase Premium • Ordnance Survey Integrated Transport Network	• Townsend deprivation index • Residential density • Retail density • Density of Transit Stops • Street movement density • Accessibility • NDVI • Mean terrain (deg.) Walkability Index	Hypertension: • DBP • SBP	Low
Dengel et al. [46] (United States)	Design: Longitudinal Sample: 188 American Adolescents, aged 10–16 Area: Buffer: Residential Address	Population: • Transdisciplinary Research on Energetics and Cancer—Identifying Determinants of Eating and Activity (TREC-IDEA) Neighborhood: • Transdisciplinary Research on Energetics and Cancer—Identifying Determinants of Eating and Activity (TREC-IDEA) • 2000 US Census, MetroGIS, • 2000 Generalized Land Use • Counties, Cities and Towns • MetroGIS Regional Parcel Dataset • 2000 Land-Use Data (Water Features data only) • 2000 Census Geography data	• Land-use Mix • Street Pattern • Employment Density • Population Density • Distance to: 1. Transit Stops 2. Fast Food outlets 3. Food Retail 4. Parks 5. Non-Fast Food outlets 6. Grocery Stores 7. Large Grocery stores 8. Convenience/Gas 9. Gym 10. Recreation 11. Centre 12. Walking/Biking Trail • Density of: 1. Non-Fast Food outlets 2. Intersections 3. Fast food outlets 4. Grocery Stores 5. Large Grocery stores 6. Convenience/Gas, 7. Busy Streets 8. Transit Stops 9. Residential areas • % Land-Use for: 1. Residential 2. Parks and Recreation 3. Vacant/unused	Metabolic Syndrome: • % Body Fat • HDLC • SBP • Weight • Fasting glucose • Triglycerides	Moderate
Li et al. [47] (United States)	Design: Longitudinal Sample: 1,145 American adults aged 50–75 at baseline, from Portland Area: Census Tract	Population: • Portland Neighborhood Environment and Health Study • 2000 Census data Neighborhood: • Infousa.com Regional Land Information System data	• Median Household Income • Land-use Mix • Street Connectivity • % of non-Hispanic black residents • % of Hispanic residents • Density of: 1. Transit Stops 2. Greenspace 3. Public Open Space 4. Fast Food outlets 5. Residential areas	Blood Pressure: • SBP • DBP	Low
Mancus et al. [44] (United States	Design: Cross-sectional Sample: 84 American adults aged 18–44, from Baltimore Area: Buffer: Residential Address, Buffer: Residential Blocks	Population: • ESSENCE Project Neighborhood: • US Geological Survey (USGS) of Baltimore	• NDVI • Crime rate • % of Vacant Properties • Daily vehicles count • Population Density	DHEA/Cortisol ratio: • Cortisol • DHEA	High
Chai et al. [45] (United States)	Design: Cross-sectional Sample: 6,643 American adults aged 19–65 Area: Census Tract, County Level	Population: • National Health and Nutrition Examination Survey (NHANES) Neighborhood: • 2000 Census data • CDC Research Data	• Safety • Crime Rate • Income to Needs Ratio • Urban Area • Tract socioeconomic status • Tract USDA food desert status	Inflammation: • Serum 25(OH)D • CRP	Moderate
Zhang et al. [43] (United States)	Design: Longitudinal Sample: 160,000 American Adults diagnosed with diabetes, aged 19+, with a mean age of 63 (SD = 12.6) Area: Network Distance, Buffer: Census Block Centroid	Population: • Kaiser Permanente Northern California Diabetes Registry Neighborhood: • InfoUSA business establishment data Standard Industrial Codes (SIC) of businesses	• Change in supermarket presence • Physical activity kernel density • Unhealthful food outlet kernel density • Population Density • % Population in poverty • House Valuation • Comorbidity Index	• HbA1c	High
Knobel et al. [42] (United States)	Design: Longitudinal Sample: 377 American Adults aged 18+, from Pennsylvania Area: Census Tract	Population: • Southeastern Pennsylvania Household Health Survey (SEPAHH) Neighborhood: • Moderate-resolution Imaging Spectroradiometer (MODIS) of NASA’s Terra satellite	• Population density • % of non-Hispanic black residents • % Population in poverty • % Vegetation Cover • % Tree canopy cover • NDVI	• Hypertension	High
Baumgardner et al. [41] (United States)	Design: Cross-sectional Sample: 931 American Adults aged 18+ Area: Network Buffer: Residential Address	Population: • Hospital-based family medicine residency clinic • Community family medicine clinic Neighborhood: • AGS Freeway 3.0 Drive Time Systems	• Distance to Health Facility	• Blood Pressure	High
Christine et al. [40] (United States)	Design: Longitudinal Sample: 5,124 American Adults aged 45–84 Area: Buffer: Residential Address, Census Tract	Population: • Multi-Ethnic Study of Atherosclerosis (MESA) Neighborhood: • 2000–2012 National Establishment Time Series database • 2000 U.S. Census • 2005–2009 and 2007–2011 American Community Survey (ACS)	• Healthy Food Environment score • Physical Activity score • Area Socioeconomic Status Social Environment score • Density of: 1. Supermarkets 2. Commercial Recreational Establishments	• Fasting Blood Glucose	Moderate
Geraghty et al. [39] (United States)	Design: Cross-sectional Sample: 7,288 American Adults, with a mean age of 62 (SD = 14.08) Area: Buffer: Residential Address, Linear Distance, Census Tract	Population: • University of California Davis Health System’s electronic medical record system Neighborhood: • 2008 Census Bureau data • 2000 census tracts	• Area Socioeconomic Status • Distance to Health Facility	• HbA1c	High
Lee et al. [38] (United States)	Design: Cross-sectional Sample: 570,645 American Adults aged 18+ Area: Census Tract	Population: • NYC A1C Registry data Neighborhood: • 2009–2013 American Community Survey (ACS) • Statewide Planning and Research Cooperative System Database (SPARCS)	• % of non-Hispanic black residents • % Population in poverty • Area-level education • Median Household Income • Diabetes-specific inpatient hospitalizations • Diabetes-specific emergency visits • Diabetes Prevalence	• HbA1c	Moderate
Kahr et al. [37] (United States)	Design: Cross-sectional Sample: 80 pregnant American women with a mean age of 29 (IQR = 24.3—33.9) Area: Buffer: Post Codes, Zip Codes	Population: • PeriBank Neighborhood: • 2011 and 2012 US Census	• Population Density • Density of: 1. Food Establishments 2. Fast Food, 3. Supermarkets	• HbA1c	High
Egorov et al. [48] (United States)	Design: Cross-sectional Sample: 206 American adults, with a mean age of 37 and a median age of 33 Area: Buffer: Residential Address, Census Tract, Buffer: Residential Blocks	Population: • Data from a previously conducted study in the Durham-Chapel Hill, North Carolina metropolitan area Neighborhood: • EnviroAtlas	• Density of: 1. Greenspace 2. Residential areas	Allostatic Load: • CRP • Fibrinogen • Uric Acid • HDLC • Cholesterol • LDLC • Epinephrine • Norepinephrine • Dopamine • interleukin (IL)−1β • IL-6 • IL-8 • TNF • SAA • VCAM-1	High

HDLC, High-Density Lipoprotein Cholesterol; LDLC, Low-Density Lipoprotein Cholesterol; HbA1c, Glycated haemoglobin; SBP, systolic blood pressure; DPB, diastolic blood pressure; BMI, body mass index; CRP, C-Reactive Protein; NDVI, normalized difference vegetation index; DHEA, dehydroepiandrosterone; CDC, centers for disease control and prevention; IQR, interquartile range; TNF, tumor necrosis factor; SAA, Serum amyloid A; VCAM-1, Vascular cell adhesion molecule 1.

### Data Sources

Most studies used different sources of population data. Only the Prospective Urban Rural Epidemiology [29, 35] study and the Montreal Neighborhood Survey of Lifestyle and Health [27, 30] were used twice, making direct comparisons difficult. Ten studies [26, 28–30, 34, 35, 38, 45–47] used nationally representative data from surveys and panel studies, five [29, 32, 34, 35, 44] used cohort data, four [38, 39, 41, 43] used administrative health data, and two studies each used government [32, 47] and/or commercial [36, 37] databases. Two studies used data from an opportunity sample [33, 48]. All the included studies relied on different sources of neighborhood data in addition to population data sources. Census data was the most commonly used data source for neighborhood-level data with twelve studies using this [26–31, 37–40, 45, 46]. Transport [27, 30, 35, 40, 45], business type [26, 30, 32, 43, 47], and satellite [29, 34, 42, 46] data were also used by multiple studies to create GIS measures of neighborhood contexts.

### Sample/Population

Ten studies [26, 28–30, 34, 35, 38, 45–47] used a representative design. In addition, three studies used extremely large samples ranging from 160,000 to 570,645 [36, 38, 43], with two using diabetes registry data [38, 43], although these studies did not use representative designs. Twelve studies [26, 28, 29, 31–34, 39–41, 45, 47] had large samples ranging from 931 to 7,288 and eight [27, 30, 35, 37, 42, 44, 46, 48] used small samples ranging from 80 to 377.

### Areal Units

Five studies used census tract or city neighborhood boundaries as the lowest areal unit of their analysis [32, 38, 42, 45, 47]. Eighteen studies [26–31, 33–37, 39–41, 43, 44, 46, 48] used residential addresses as the lowest areal unit, which required calculating single or multiple buffer areas around point data. Buffers refer to the specific areas within boundaries drawn depending on a given criteria, with boundaries typically applied based on distance, time, or relative to administrative boundaries. Where studies considered residential address as the lowest areal unit, network distances were used to calculate buffer boundaries. For example, how many healthcare centers could be reached by paths and/or roads in 500-meter or 10-minute walking time from residential address. One study additionally constructed straight-line radial buffers, which are calculated by measuring x-meters in every direction around an address, indicating “as the crow flies” distance without accounting for potential barriers [46]. One study also compared school *versus* home address [34] as the lowest areal unit around which to calculate buffers, and one considered the spatial extent of all adjacent postcodes as buffer boundaries [37]. Thirteen studies calculated multiple buffer areas in order to assess the relative importance of their exposures across different scales [26, 29, 31, 33–36, 38, 40, 41, 44, 46, 48], with 10 studies using a single buffer or an administratively defined areal unit [27, 28, 30, 32, 37, 39, 42, 43, 45, 47]. Multiple buffers were used to answer questions about the relative importance of proximal access to a given feature or resource, such as a community center, within 500-meter of a residential address compared to within 1,000-meter, or the importance of having x-amount of a given resource within 500-meter compared to 1,000 m.

Where buffer analysis was used to create boundaries around residential addresses that crossed administrative areas, studies estimated weights to account for areas overlapping pre-defined boundaries, such as census tract, to create estimated population levels based on area covered across each administrative boundary [27, 29, 38]. Buffers based on distances ranged from a low of 100-meter through to a high of just under 5,000-meter, with 500-metre buffers being the most commonly considered areal unit. Where buffers were calculated based on time [26, 29], buffers were calculated based on incremental increases to estimated walking time in bands of 4 or 5 min up to a maximum buffer considered of greater than 30-minute.

### GIS Measures

114 unique GIS measures were identified across included studies, of which 78 were independently tested against stress and AL-related biomarkers. Of these 78 measures, 64 were measures of density (how much of a given resource within a given boundary) and 14 were measures of proximity (how far to specific resources in distance or time). Across these measurement types, broad themes existed in terms of what aspects of the neighborhood context were being explored, with 25 measuring greenspace, 24 GIS measures capturing aspects of built environment land-use, 19 assessing area-level demographics, and 10 the food environment.

GIS measures of greenspace captured: greenspace coverage [29, 32, 36, 42, 44, 48], number of accessible greenspaces [34]; greenspace variability [29]; greenspace quality [29]; the relative importance of proximal access to greenspace from school or residential address [34]; the distance to the nearest greenspace without a pre-defined boundary [34]; distance to public open spaces, defined as publicly owned spaces larger than 700-meter squared, with or without provision for recreational activities, including nature reserves [26]; and differences between perceived and objective distances to public open spaces [26] (see Table 2). One study created a composite index of diabetes risk, where high index values corresponded to socially deprived areas with low levels of greenspace [29]. The Normalized Difference Vegetation Index (NDVI) was the most frequently used measure of greenspace exposure, used by five studies [29, 36, 42, 44, 48], although it was not uniformly constructed (in terms of resolution or area) or considered (in terms of expected effect). NDVI has scores ranging between −1 and 1 with distinct boundaries for types of land cover and measures the difference between near-infrared bandwidths (NIR) and visible red (RED) bandwidths [40]. Negative values are likely to be water, scores close to zero are likely to be urban areas or grey-space (without green), and scores close to 1 are likely to be densely green. Two studies used a similar measure of Greenspace as NDVI defined using Light Detection and Ranging (LiDAR) [42, 48]. LIDAR is a remote sensing technology that uses laser light to generate three-dimensional information about the Earth’s surface, including vegetation among others. Using LIDAR one can obtain geo-referenced data of the tree-canopy and then estimate the proportion of space covered by this canopy, as well as data on their density and height. Land covered by green urban spaces [32]; land covered by sports facilities [32]; having a garden [34]; and the number of green spaces accessible within a given radius around the household or school [34]. In each case, higher levels of greenspace were predicted to be salutogenic, regardless of the outcome considered.

**TABLE 2:** Description of Geographic Information Systems measures (systematic review, worldwide, journal start-date-2022).

GIS Measures	Type	Use of Buffers	Commonalities and Differences
Area level demographic			
% Dwelling Ownership [29]	Density		All but 2 GIS measures of area-level demographics were measures of density, with the other 2 being a combination of density measures within an index score. These measures typically accounted for population size within administrative units, with existing boundaries being used to aggregate measures of exposure. However, one measure calculated a weighted average of the unemployment rate for census tracts over which a 250-meter resident centered buffer overlapped, with weights corresponding proportionately to the overlap area. Multiple buffer areas were not considered by any measures of this type except for the study using hot-spot analysis. Measures used in the hot-spot analysis study were mapped using a 1.5 miles distance band based on Getis-Ord General G statistic, although sensitivity analysis was conducted to test the effects of using other distance bands. Two studies used factor analysis to create census-tract level measures of socioeconomic status using a range of area-level demographic measures adjusted for population size
% Dwellings Rented [29]	Density		
Crime rate [45]	Density		
Education - no degree (%) [29]	Density		
Estimated age-adjusted diabetes prevalence [38]	Density		
Frequency of diabetes-specific emergency visits [38]	Density		
Frequency of diabetes-specific inpatient hospitalizations [38]	Density		
Labour force participation rate (%) [29]	Density		
Lone Parent Families (%) [29]	Density		
Prevalence of Low Income (%) [29]	Density		
Proportion of Hispanic residents [38]	Density		
Proportion of minority residents [38]	Density		
Proportion of non-Hispanic black residents [38]	Density		
Proportion of residents below poverty level [38]	Density		
Proportion of residents < high school degree or equivalent [38]	Density		
Socioeconomic Status of Area (4 components) [39]	Index		
Tract SES factor score (3 Components) [45]	Index		
Unemployment rate (weighted) [27]	Density	X	
Unemployment rate (%) [29]	Density		
Food Environment			
Change in supermarket presence [43]	Density	X	Measures of the food environment essentially represented a specific form of built environment land use. Ten measures were density measures, 2 were proximity measures, and one was an index score. Three studies calculated density by population size, with rates of the food environment calculated per 100,000 of the population in 2 studies. Food deserts were defined as census tracts with at least 500 residents or 33 percent of a population living far from a large supermarket or equivalent. Buffers were calculated for both distances and time, ranging from 500 m–4,828 m and 0 to >30 min walking time respectively. A composite score was calculated that incorporated objective and subjective availability of healthy food. Two studies also considered changes in the configuration of the food environment
Density of fast-food restaurants [30, 47]	Density	X	
Density of supermarkets and Fruit and Vegetable markets [40]	Density	X	
Discordance between perceived and objective measures of Food and Vegetable Retailers [26]	Proximity	X	
Distance to Food and Vegetable Retailers [26]	Proximity	X	
Fast Food Restaurant per Supermarket ratio [37]	Density		
Fast food restaurants per 100,000 inhabitants [37]	Density		
Healthy Food Environment Summary Score [40]	Index	X	
No. of street markets per week [32]	Density		
No. of supermarkets (per 100,000 population; by 10 markets per 100,000 population increment) [32]	Density		
Tract USDA food desert (vs non-food desert) [45]	Density		
Greenspace			
Discordance between perceived and objective measures of distance to Public Open Space [26]	Proximity	X	Sixteen measures of greenspace were density calculations, 5 considered proximity, and one was an index score. NDVI was the most commonly used measure of greenspace. However, whilst the NDVI has scores ranging between −1 and 1 with distinct boundaries for types of land cover and measures the difference between near infrared bandwidths (NIR) and visible red (RED) bandwidths, various specificity was used in terms of pixel values of spectral reflectance, ranging from 0.50 cm to 250-meter across studies. Consideration was also given to the importance of greenspace around home and school addresses as well as the importance of presence, amount, and proximity of greenspace. No measures of greenspace accounted for population size of the areas measured. Only 2 greenspace measures used buffers rather than pre-existing administrative units to define boundaries within which to calculate exposures. Buffers calculated included network measures of distance and time, ranging from “50-m” through to “greater than 2,400-m” and “0” through to “greater than 30-minute” in walking time. The index score was calculated using principal component analysis and combined 4 measures of greenspace with 11 measures of area-level socioeconomic status
Distance to Public Open Spaces [26]	Proximity	X	
Distance to the nearest green space [34]	Proximity	X	
DRI-GLUCoSE Index	Index	X	
Green space presence near school [34]	Proximity	X	
Green space presence neare residence [34]	Proximity	X	
Home garden [34]	Density	X	
Land covered by green urban spaces	Density		
Land covered by sports facilities	Density		
LIDAR - Light Detection and Ranging [48]	Density	X	
NDVI—Min [29]	Density	X	
NDVI—Max [29]	Density	X	
NDVI—Mean Quartiles [36]	Density	X	
NDVI—median [29]	Density	X	
NDVI—Normalized Difference Vegetation Index [44]	Density	X	
NDVI—Overall Greeness	Density		
NDVI - Standard Deviation [29]	Density	X	
No. of green spaces near school [34]	Density	X	
No. of green spaces near residence [34]	Density	X	
Perceived access to green space (% of adults reporting)	Density	X	
Percent tree canopy cover (%)	Density	X	
Percent vegetation cover (%)	Density	X	
Land Use			
Density of Commercial recreational establishments	Density		Measures of the built environment grouped under land use were varied and included some of the more complex measures in terms of calculation and computation. This allowed for consideration of accessibility that went beyond the presence, amount, or distance of features, towards objectively measuring potential physical barriers within the built environment through the use of network analysis. Eight measures were of density, 7 were of proximity, and 6 were indices. There were 15/21 measures that used buffers when constructing this type of measure. Buffers considered ranged from “500-m” to “>68,000-m”, and 4-minute walking time thresholds ranged between 0 and 20 min. As well as basic distance and density measures, some measures sought to capture the topology and connectivity of locations using measures of GIS that calculated connectivity of streets around home addresses and towards specific features of the built environment deemed to facilitate physical activity. However, connectivity was measured differently across the studies using this type of measure (see Supplementary Material for a detailed breakdown of how each individual measure was calculated). A number of indices sought to capture the overall walkability of the built environment factoring in a number of measures relating to land-use of the built environment. Again, these measures shared some components, such as connectivity, terrain slope, and land-use diversity, but similar types of measures were not uniformly calculated. For example, some measures accounted for area based on actual land-mass, or did or did not account for population size
Density of Retail	Density			
Destination accessibility (mean street network distance)	Proximity			
Distance to Community Centre	Proximity			
Distance to convenience/gas station network	Proximity		
Distance to Shopping Centre	Proximity		
Distance to Taxi Rank	Proximity		
Driving Distance to Clinic	Proximity		
Neighborhood active-living environment index	Index		
Physical Activity Summary Score	Index		
Public Transport Density	Density		
Residential Density	Density		
Road Network Distance	Proximity		
Slope variability	Density		
Street movement density	Density		
T”he WS (a measure of walkability)	Index		
Tract urban area (vs. rural area)	Density		
Urbanity level	Density		
Walkability Index 1	Index		
Walkability Index—Quartiles	Index		
Walkability Index 2	Index		

Area-level demographic density measures considered: crime [45]; education level [29, 38]; employment [27, 29]; ethnicity [38]; housing tenureship [29]; and socioeconomic status (SES) [29, 38, 39, 45]. All studies including area-level demographic density measures accounted for population levels, whereby the proportion of each measure was relative to population size across areas considered, such as county crime rate by 1,000 people. One study used hot-spot analysis (a spatial analysis technique used to assess whether high or low values cluster in a way that appears to be geographically patterned) to test the predictive ability of indirect measures against an HbA1c registry and found: diabetes-specific inpatient hospitalizations (accuracy: 89%); diabetes-specific emergency visits (90%), age-adjusted diabetes prevalence estimated from emergency department data (89%); and the proportion of minority residents (86%), as the highest-performing predictors compared to an HbA1c registry, with accuracy calculated as (true positives + true negatives)/all observations. Proportions of non-Hispanic black residents, residents below the poverty level, residents with less than a high school degree or equivalent, and Hispanic residents all had poorer (<80%) accuracy ratings using this approach [38].

Food environment measures sought to represent positive or negative exposures relating to food retailers available in an area. Supermarket presence [43], proximity to food and vegetable retailers, higher densities of street markets [32] and supermarkets [32, 37, 40, 43], and a composite index to measure the healthiness of the food environment which combined objective and perceived measures of healthy food access [40] were predicted to have beneficial effects on outcome measures, with fast food outlet density [30, 37, 47], discordance between perceived and objective distance to food and vegetable retailers [26], and food deserts [45] theorized to have negative effects on outcomes. Measures of land-use captured: public transport access [36]; access to commerce and recreation [36, 40]; urbanity-level [29, 45]; residential density [36]; area level physical accessibility [36]; distance to community centers [35]; distance to shopping centers [35]; distance to convenience stores [46]; distance to taxi ranks [35]; distance to healthcare [41]; and distance to population centers [33]. One measure assessed overall destination accessibility by calculating the average distance to schools, medical facilities, leisure, retail and places of worship within their defined buffer [36]. Six studies [28, 29, 31, 36, 40, 47] used multiple GIS measures to create an index, with included measures sometimes crossing the overarching sub-themes and measurement types. These indices sought to capture: walkable environments [28, 31, 36, 40, 47]; healthy food environments [40]; and diabetes risk [29].

### Measuring Density With GIS

All but 2 [33, 41] of the 23 included studies used a GIS measure of density to assess the neighborhood context and the composition of the built environment. Density measures were calculated based on how much of a particular resource or feature there was relative to a given area [29, 30, 32, 34, 36, 40, 42–44, 47]; area and population [27, 29, 32, 37–39, 42, 45]; area, population, and time [32]; and area relative to another food environment feature [37, 45].

### Measuring Proximity With GIS

Seven studies [26, 33–36, 41, 46] used a GIS proximity measure. All used road network buffers rather than radial buffers when calculating proximity measures. However, one study [46] also considered straight line (“as the crow flies”) distance. Unlike network distances, straight-line distances ignore any barriers (such as fencing/entry-points/motorways) that might exist between points. All studies using proximity measures used residential address as the start point from which to calculate distances to features of the neighborhood context, they theorised to impact their outcome. One study [34] also considered distances around the school in order to consider the impact of activity spaces around both the home and school environments of their sample of school-aged children. One study [26] first calculated distance, then estimated the walking time based on average walking speeds.

### Outcome Measures: Biomarkers and Allostatic Load

Table 3 lists the 33 outcomes tested for an association with the GIS measures, indicating whether any significant (*p* < 0.05) associations were found or not for area-level demographic, food environment, greenspace, and land-use measures. Only two studies [34, 48] measured AL as an outcome and the biomarkers included within each measure of AL differed across the studies. Consequently, studies wishing to tease out the effects of GIS-measured neighborhood effects on AL may find this variation to be a barrier to effective interpretation of effects. Similarly, three [26, 32, 46] of the four studies [26, 30, 32, 46] that assessed the Metabolic Syndrome as an outcome conflated the included biomarkers into a composite measure obscuring individual biomarker relationships. However, these studies were included as several of the biomarkers used to measure Metabolic Syndrome, such as triglycerides, cholesterol, and hemoglobin A1c (HbA1c), are commonly used within measures of AL [49].

**TABLE 3:** Biomarker associations with Geographic Information Systems measures of the neighborhood context (systematic review, worldwide, journal start-date-2022).

Outcome measure	Area-level demographic	Food environment	Green-space	Land-use	Tested GIS Measure/s significantly associated (*)/Not significantly associated (NS)
α-amylase			NS		(NS) LIDAR
Allostatic Load			* (& NS)		(*) Green-space 400/800 m from school (yes), nearest (km), LIDAR
					(NS) Number greenspaces 400/800 m from school/residence, greenspace 400/800 m from school (yes), home garden (yes)
BMI	*	Papers focused solely on BMI and food environment were excluded	*	* (& NS)	(*) % unemployed, green-space 800m from school, distance to Community Centre/Shopping Centre, active-living environment index, Walkability Index 2, The WS
					(NS) Distance to Taxi Rank, Density of fast-food restaurants 500 m
Body Fat (%)				NS	(NS) Distance to convenience/gas station network
CRP	* (& NS)	NS	NS	NS	(*) County crime rate (# of crime/1,000 persons)
					(NS) Green-space at 400/800 m from school/residence (yes), Number greenspaces 400/800 m from school/residence, Distance to nearest green space (km), garden (yes), Tract urbanity/SES factor score/USDA food desert, LIDAR
DHEAS			*		(*) LIDAR
DHEA/Cortisol Ratio			*		(*) NDVI
Diastolic Blood Pressure	* (& NS)	* (& NS)	* (& NS)	* (& NS)	(*) Distance to Community Centre, Destination accessibility, Walkability Index, Density of fast-food/Retail/Public Transport/Street movement/residential, Slope variability, NDVI, Walkability Index, Walkability Index 2, The WS
					(NS) Distance to Shopping Centre/Taxi Rank/Food and Vegetable Retailers/Public Open Spaces/green-space, Road Network Distance, Green-space number of/at 400/800 m from school/residence (yes), garden, Discordance perceived vs. objective Public Open Space/Food and Vegetable Retailers, Walkability Index
Dopamine			NS		(NS) LIDAR
Epinephrine (Adrenaline)			*		(*) LIDAR
Fasting glucose	* (& NS)	* (& NS)	* (& NS)	* (& NS)	(*) Healthy Food Environment Summary Score, Physical Activity Summary Score, DRI-GLUCoSE Index
					(NS) Distance to convenience/gas station/Food and Vegetable Retailers/Public Open Spaces, Land covered by green urban spaces/sports facilities, urbanity level, density of street markets/supermarkets/Fruit and Vegetable markets/recreational establishments, Discordance perceived vs. objective Public Open Space/Food and Vegetable Retailers, NDVI, % no degree/lone-parent/low income/home owners/renters/working/unemployed
Fibrinogen			*		(*) LIDAR
HbA1c - glycated haemoglobin	* (& NS)	* (& NS)	* (& NS)	* (& NS)	(*) Area-level unemployment, Neighborhood active-living environment index, Change in supermarket presence, Socioeconomic Status of Area, Fast Food Restaurant per Supermarket ratio
					(NS) Green-space number of/at 400/800 m from school/residence (yes), garden, Walkability Index 2, The WS, Distance to green-space, Density of fast-food restaurants, % non-Hispanic black residents/Hispanic residents/below poverty level/minority residents/with <high school/diabetic, Frequency of diabetes- hospitalizations/emergency visits
HDLC—High-Density Lipoprotein Cholesterol Level	* (& NS)	NS	* (& NS)	NS	(*) LIDAR, Area-level unemployment
					(NS) Green-space number of/at 400/800 m from school/residence (yes), garden, Land covered by green urban spaces/sports facilities, density of street markets/supermarkets/fast-food, Walkability Index 2, The WS, Distance to Food and Vegetable Retailers/Public Open Spaces/gas station/green-space, Discordance perceived vs. objective Public Open Space/Food and Vegetable Retailers
Hypertension	* (& NS)	*	* (& NS)	* (& NS)	(*) Distance to Public Open Spaces/Food and Vegetable Retailers, Discordance perceived vs. objective Public Open Space/Food and Vegetable Retailers, Walkability Index—Quintiles, Walkability Index 2, % Vegetation Cover
					(NS) % Perceived access to green space/tree cover/vegetation cover, NDVI, Road Network Distance, Density of Residential/Retail/Public Transport/Street movement, Destination accessibility, Slope variability, Walkability Index, The WS
ICAM-1 –Intercellular adhesion molecule 1			NS		(NS) LIDAR
IL−1β Interleukin 1β			NS		(NS) LIDAR
IL-6—Interleukin 6			NS		(NS) LIDAR
IL-8—Interleukin 8			NS		(NS) LIDAR
LDLC - Low Density Lipoprotein Cholesterol	NS		NS	NS	(NS) Walkability Index 2, The WS, LIDAR
Metabolic Syndrome		* (& NS)	* (& NS)	* (& NS)	(*) Distance to convenience/gas station network, Land covered by green urban spaces/sports facilities, No. of street markets/supermarkets
					(NS) Distance to Food and Vegetable Retailers/Public Open Spaces, Discordance perceived vs. objective Public Open Space/Food and Vegetable, Density of fast-food
Myeloperoxidase			NS		(NS) LIDAR
Norepinephrine (Noradrenaline)			*		(*) LIDAR
Serum amyloid A			NS		(NS) LIDAR
serum 25(OH)D levels	* (& NS)	NS		NS	(*) County crime rate (# of crime/1,000 persons)
					(NS) Tract urbanity/SES factor score/food desert (vs non-food desert)
Systolic Blood Pressure	* (& NS)	* (& NS)	* (& NS)	* (& NS)	(*) Distance to Shopping Centre, Density of fast-food/Residential/Retail/Public Transport/Street movement, Destination accessibility, Slope variability, NDVI, Walkability Index, Walkability Index 2, The WS, Active-living index
					(NS) Distance to Community Centre/Taxi Rank/gas station/green-space/Food and Vegetable Retailers/Public Open Spaces, Discordance perceived vs. objective Public Open Space/Food and Vegetable, Road Network Distance, Green space at 400 m from school (yes), Green-space number of/at 400/800 m from school/residence (yes), garden, Walkability Index
Total Cardiovascular Risk	*				(*) Area-level unemployment
Total cholesterol	*	* (& NS)	* (& NS)	* (& NS)	(*) Area-level unemployment, active-living index
					(NS) Green space at 400 m from school (yes), Green-space number of/at 400/800 m from school/residence, garden, distance to green-space, Density fast-food
Total cholesterol to HDL-C ratio		NS	NS	NS	(NS) Neighborhood active-living environment index
Triglycerides	*	NS	NS	NS	(*) Area-level unemployment
					(NS) Distance to gas station/Food and Vegetable Retailers/Public Open Spaces, Discordance perceived vs. objective Public Open Space/Food and Vegetable, Land covered by green urban spaces/sports facilities, Number of street markets/supermarkets, Density of fast-food, Walkability Index 2, The WS

* indicates when a significant association was found (*p* < 0.05), NS indicates when non-significant associations were found, * (& NS) indicates mixed findings, LIDAR, light detection and ranging; NDVI, normalized difference vegetation index.

Where associations with individual biomarkers were reported with a GIS measure: 9 studies included systolic blood pressure (SBP) [26, 28, 31, 33–36, 46, 47]; 9 HbA1c [28, 31, 33–36, 43, 46, 47]; 8 high-density-lipoprotein-cholesterol (HDL-C) [26, 27, 30–32, 34, 46, 48]; 7 diastolic blood pressure (DBP) [26, 31, 33–36, 47]; 6 triglycerides [26, 27, 30–32, 46]; 5 BMI [27, 28, 30, 31, 35]; 5 fasting glucose [26, 29, 32, 40, 46]; three waist circumference [26, 30, 32]; 3 C-Reactive Protein (CRP) [34, 45, 48]; three total cholesterol [27, 30, 34]; 2 low-density-lipoprotein-cholesterol (LDL-C) [31, 48]; 1 dehydroepiandrosterone sulfate (DHEAS) and DHEAS to cortisol ratio [44]; 1 serum 25(OH)D levels [45]; and 1 total cholesterol to HDL ratio [28]. A single study also reported associations with a GIS measure for: intercellular adhesion molecule 1 (ICAM-1); vascular cell adhesion molecule 1 (VCAM-1); interleukin-6; interleukin-8; interleukin-1β; myeloperoxidase; tumor-necrosis factor (TNF); serum amyloid A; uric acid; and α-amylase [48].

#### Assessment of Risk of Bias

The overall quality of evidence was found to be moderate-to-low quality for the purpose of assessing the relationships between neighborhood characteristics and AL. Six of the identified studies [26, 28, 31, 34, 36, 47] were rated as having a low risk of bias, eight demonstrated a moderate risk of bias [27, 29, 32, 35, 38, 40, 45, 46] and nine were determined to have a high risk of bias [30, 33, 37, 39, 41–44, 48]. As all study types were eligible for inclusion, a specific JBI appraisal tool was not available for all of the included studies. However, to assess each included study consistently the checklist for analytical cross-sectional studies appeared most suitable [25]. The main concerns for studies with a high risk of bias related to issues with sample size and sample selection relative to the analysis being undertaken. Other factors that led to moderate or high-risk scores included the lack of strategies being identified to deal with confounding factors, including classic issues in GIS research, such as date discrepancies between linked data, the modifiable areal unit problem (MAUP), the uncertain geographic context problem (UGCoP), and the issue of self-selection into neighborhoods.

## Discussion

This systematic review was the first to consider how GIS has been used to measure relationships between access to the built environment and biomarkers of stress and AL, and to assess the quality of the existing evidence to address this question. It highlighted there is limited research in this area, with only 2 of the 23 included studies considering AL as an outcome directly. However, relationships between neighborhood attributes and relevant measures of the stress pathway were evident in this review, with findings pointing to this area being an interesting avenue for future research. For example, both studies that tested for an association between greenspace and AL, found greenspace impacted overall AL. Moreover, each tested associations at different scales using multiple buffers. This approach addresses two common issues in GIS research, described as the modifiable areal unit problem (MAUP) and the uncertain geographic context problem (UGCoP) [50]. The MAUP relates to the fact that the effects of area-based variables may be affected by the scales being used and ecological fallacy whereby group characteristics are ascribed incorrectly to individuals [50]*.* Whilst the UGCoP arises due to uncertainty surrounding what areas exert the most influence over individuals and uncertainty surrounding actual exposures to different contexts [50]. The UGCoP was particularly relevant in the Portuguese study, where they found a significant association between access to greenspace in the area around schools and AL, but not around the home address, for their school-aged sample. In this case, the school context appeared more important for the primary school-aged participants in terms of greenspace exposure and AL. Whilst few of the included studies explicitly addressed the MAUP and the UGCoP, the use of multiple buffers, sensitivity analysis, and studies that used longitudinal designs partially addressed these concerns. Whilst studies focused on GPS and accelerometer data were not retrieved and retained due to their individual level focus, measures calculated using this technique are argued to directly address concerns related to the UGCoP by tracking individual activity spaces. For example, this approach often centers analysis around the aspects of the built environment individuals actively engage with [51, 52]. This would be an interesting addition to the literature surrounding neighborhood access to the built environment and Allostatic Load, however, as the focus of this approach shifts from what features of the built environment are accessible at the neighbourhood level within a given space to how this space is used at a more individual level, studies of this type were not included.

The review highlights a lack of consistency in the GIS measures being used, with few studies using any of the same measures. However, broad themes were evident in terms of how measures were calculated (distance/density) and the aspects of the built environment the GIS measures sought to capture (area-level demographics, land-use, greenspace, and the food environment). Nevertheless, even where measures were ostensibly similar, such as with measures of walkability, land-use-mix, and NDVI, they were often calculated differently, whether by the collation of varied measures in indices, or by using different degrees of specificity in terms of raster imagery, buffer sizes, and/or weighting schemes, making comparisons difficult. These variations in construction may explain conflicting results across studies, but few studies have sought to directly compare NDVI with other measures of greenspace [53]. Nevertheless, differences in measures were often due to data availability, and in part reflects an advantage of using a GIS approach, in that a broader array of data sources can be brought together to deepen insight into the nature of how individuals are exposed to complex neighborhood environments. However, when different data sources are drawn together, there is the potential for a time lag between any measurement of exposure and outcome that may influence findings and interpretations, increasing the risk of bias. For example, Zhang et al. noted that the time interval between their calculation of change of supermarket presence and assessment of participant HbA1c ranged from 1 day up to 24 months. Given associations were short-lived and were not observed in long-difference regression models, this likely influenced results. As such, more work needs to be done to identify suitable techniques to manage analysis of disparate datasets collected over differing time-periods.

A noted strength of GIS measures relates to data quality and objective measurement. However, the inclusion of both objective and subjective measures in several studies also made it possible to consider the reliability of findings, and test the effectiveness of both GIS and subjective measures. For example, Baldock et al., found a subjective measure of perceived distance to healthy food environments was associated with increased hypertension risk [OR: 1.13 (CI: 1.02, 1.25), p. 0.005], but objective distance was not. However, combining this with the GIS measure made it possible to identify participants who overestimated distances, with this group having an increased risk of hypertension [OR:1.36 (CI: 1.02, 1.80) p. 0.034]. A plausible explanation for this is that people who overestimate distances may have factored in barriers to their estimation, which might be ignored by a simple objective measure of distance. As such, studies using GIS measures should consider subjective measures that might complement, expand on, and explain how access to the built environment may impact exposure to environmental stressors in greater detail (and *vice versa*).

### Limitations

Owing to the highly varied language used surrounding GIS and the lack of consistency in how AL is conceptualized and measured [54], the broad range of search terms may still fail to pick up all of the relevant literature, particularly when combined with our exclusion criteria. For example, the decision to omit papers solely focused on BMI and not to include terms such as “GPS” and “Accelerometer” in our search terms likely impacted the types of exposures that were considered in terms of GIS measures. However, GIS measures centered on the use of Geographical Positioning System (GPS) devices tend to focus on the individual scale, rather than the effects of the neighborhood level which was the focus of this review. These types of measures have also typically been used to assess the relationship between characteristics of the built environment and activity spaces (e.g., physical exercise) [52, 55] which is the focus of a number of forthcoming systematic reviews listed on PROSPERO [56]. Nevertheless, the use of GPS tracking devices offers the opportunity to address common issues in GIS research, such as the UGCoP. Moreover, BMI is inconsistently included as a component of AL [54], the relationship between BMI and AL is not well understood [57], and the stress pathway was not expected to be central to the analysis in papers where BMI was the sole biomarker in retrieved studies. As such, we opted to continue to exclude BMI-specific papers despite the possible reduction in ability to identify some relevant literature using this type of measure. In addition, although this review is able to offer insights into how GIS measures have been used in AL research, because its focus was on the use of the methods the review is unable to speak to the effects or consequences of using different GIS measures.

## Conclusion

GIS measures of neighborhood attributes have been widely used in the literature to assess relationships with biomarkers associated with stress. However, only two studies considered AL directly. As such, the quality of the evidence to assess associations between GIS measures of access to the built environment and AL suffered due to heterogeneity in both exposures and outcomes. This was largely due to the limited number of studies included which directly addressed issues relating to the stress pathway. However, measures seeking to capture access to features of the built environment such as greenspace, the food environment, area-level demographics, and land-use mix were found to influence relevant biomarkers associated with AL and the stress-pathway point to this area as an interesting avenue for future research. Moreover, several studies considered multiple areas of exposure and highlighted the complimentary influence GIS measures can offer to neighborhood effects research, particularly when used in conjunction with subjective measures.
