# Supplementary material for: Neighborhood Access to the Built Environment and Allostatic Load: A Systematic Review of the Use of Geographic Information Systems
Source: Public Health Rev. 2024 May 23;45:1606624. doi: 10.3389/phrs.2024.1606624 (PMC11153763; doi:10.3389/phrs.2024.1606624)
Supplement: Supplementary file 1 [file Table1.pdf]

Supplementary Materials Table 1: Details of how GIS measures were calculated

**Primary Author, Year**	**GIS Measure (theme, type)**	**Variable description**
Chai et al., 2016	County crime rate # of crimes/1000 persons, (Area-Level Demographics, Density)	Community/neighborhood safety level captured by county crime rates (due to the unavailability of tract-level crime data), and refers to the total number of crimes per 1000 persons in the county.
Walker et al., 2022	Education - no degree (%), (Area-Level Demographics, Density)	Within Canadian census dissemination areas (the smallest available census area with an average population of 400–800 residents) for the census year 2006 in Canada.
Lee et al., 2018	Proportion of residents with <high school degree or equivalent, (Area-Level Demographics, Density)	American Community Survey data (2009 to 2013) were used to calculate the proportion of residents having less than a high school degree or equivalent at the census tract level.
Naimi et al., 2009	Area-level unemployment, (Area-Level Demographics, Density)	Determined from the census-based unemployment rate, and calculated for resident-centred 250-m buffers. A weighted average of the unemployment rate was calculated for census tracts over which the buffer overlapped, with weights corresponding to overlap area.
Walker et al., 2022	Labour force participation rate (%), (Area-Level Demographics, Density)	Within Canadian census dissemination areas (the smallest available census area with an average population of 400–800 residents) for the census year 2006 in Canada.
Walker et al., 2022	Unemployment rate (%), (Area-Level Demographics, Density)	Within Canadian census dissemination areas (the smallest available census area with an average population of 400–800 residents) for the census year 2006 in Canada.
Lee et al., 2018	Proportion of non-Hispanic black residents, (Area-Level Demographics, Density)	American Community Survey data (2009 to 2013) were used to calculate the proportion of non-Hispanic black, Hispanic, or minority (non-white or Hispanic) residents at the census tract level.
Lee et al., 2018	Proportion of Hispanic residents, (Area-Level Demographics, Density)	American Community Survey data (2009 to 2013) were used to calculate the proportion of Hispanic residents
Lee et al., 2018	Proportion of minority residents, (Area-Level Demographics, Density)	American Community Survey data (2009 to 2013) were used to calculate the proportion of minority residents

Lee et al., 2018	Estimated age-adjusted diabetes prevalence, (Area-Level Demographics, Density)	Used emergency department claims data to estimate the prevalence of diabetes among unique New York City adults who had visited an emergency department at least once during the study period and divided the number of these unique NYC adults in the emergency department claims data who had ever received a primary or secondary diagnosis of diabetes (prefix 250) by the total number of unique adults who had ever visited the emergency department. This estimate was then age-adjusted using four age strata (18–24, 25–44, 45–65, and ≥65 years old) following the direct method described by the Centers for Disease Control and Prevention.
Lee et al., 2018	Frequency of diabetes-specific inpatient hospitalizations, (Area-Level Demographics, Density)	Used SPARCS data for the annual number of inpatient hospitalizations with a primary diagnosis (not secondary) starting with the prefix of 250 (first three numbers/letters of the code) and divided it by the total number of estimated adults from the American Community Survey data by census tract.
Lee et al., 2018	Frequency of diabetes-specific emergency visits, (Area-Level Demographics, Density)	Used SPARCS data for the annual number of emergency department visits with a primary diagnosis (not secondary) starting with the prefix of 250 (first three numbers/letters of the code) and divided it by the total number of estimated adults from the American Community Survey data by census tract.
Walker et al., 2022	% Dwelling Ownership, (Area-Level Demographics, Density)	Within Canadian census dissemination areas (the smallest available census area with an average population of 400–800 residents) for the census year 2006 in Canada.
Walker et al., 2022	% Dwellings Rented, (Area-Level Demographics, Density)	Within Canadian census dissemination areas (the smallest available census area with an average population of 400–800 residents) for the census year 2006 in Canada.
Chai et al., 2016	Tract SES factor score, (Area-Level Demographics, Density)	Constructed based on percent affluent households, percent residents in poverty and percent college educated residents
Geraghty et al., 2010	Socioeconomic Status of Area, (Area-Level Demographics, Density)	Followed the approach of Diez Roux et al., 2001 to create a summary measure of census tract-level socioeconomic status using factor analysis that included: median income in 2008, proportion of population with a below-average education, proportion of the population that was unemployed, and the proportion of the population that was black or white.
Walker et al., 2022	Prevalence of Low Income (%), (Area-Level Demographics, Density)	Within Canadian census dissemination areas (the smallest available census area with an average population of 400–800 residents) for the census year 2006 in Canada.

Walker et al., 2022	Lone Parent Families (%), (Area-Level Demographics, Density)	Within Canadian census dissemination areas (the smallest available census area with an average population of 400–800 residents) for the census year 2006 in Canada.
Lee et al., 2018	Proportion of residents below poverty level, (Area-Level Demographics, Density)	American Community Survey data (2009 to 2013) were used to calculate the proportion of residents living below 100% of the federal poverty level at the census tract level.
Li et al., 2009	Density of fast-food restaurants, (Food Environment, Density)	Density score was calculated by dividing the number of fast-food restaurants by square miles within each neighborhood .
Chai et al., 2016	Tract USDA food desert (vs. non-food desert), (Food Environment, Density)	Determined using USDA Food Desert Locator, which defined food deserts as low-income tracts with at least 500 people or 33 percent of residents in the tract living far away from a supermarket, super centre, or a large grocery store.
Baldock et al., 2018	Distance to Food and Vegetable Retailers, (Food Environment, Distance)	Road network distance measured from residential address to the nearest Fruit and Vegetable Retailer (FVR) expressed as walking time (in minutes) based on a moderate adult walking speed of 4.8 km (3.0 miles) per hour.
Baldock et al., 2018	Discordance between perceived and objective measures of Food and Vegetable Retailers, (Food Environment, Distance)	Overestimation of the actual distance to FVR represented discordance between perceived and objective distances. The difference between perceived and objective distance scores was calculated as objective less perceived distance. Positive difference scores indicate underestimated distances to destinations, whereas negative difference scores indicate overestimated distances (i.e., respondents perceived the distance to destinations to be further than objective distance).
Zhang et al., 2017	Change in supermarket presence, (Food Environment, Density)	Retail locations of supermarkets and large grocery stores mapped for January 1st in each year of study period to create an indicator of supermarket presence within-1-mile street network buffer around the census block centroid of residential address. Change in neighborhood presence of supermarkets compared with the previous year was then transformed into 3 mutually exclusive indicator variables: supermarket gain, no change, and supermarket loss.
Christine et al., 2015	Healthy Food Environment Summary Score, (Food Environment, Composite Measure)	A composite score of: 1) No. Of food stores likely to sell healthier foods (supermarkets, FV markets) per square mile, with densities mapped annually to reflect changes over time/address changes &; 2) survey of perceived availability of health food stores in neighborhood (Likertscale 1-5, higher values represent more favourable environment)

Christine et al., 2015	Density of supermarkets and Fruit and Vegetable markets, (Food Environment, Density)	Number of food stores likely to sell healthier foods (supermarkets, fruit and vegetable markets) per square mile with densities mapped annually to reflect changes over time or if a participant moved.
Kahr et al., 2016	Fast food restaurants per 100,000 inhabitants, (Food Environment, Density)	Publicly available data sources including the 2011 and 2012 United States Census Bureau were utilized to determine the number of fast-food establishments per 100,000 inhabitants) on a zip code basis.
Kahr et al., 2016	Fast Food Restaurant per Supermarket ratio, (Food Environment, Density)	Publicly available data sources including the 2011 and 2012 United States Census Bureau were utilized to determine the population density (number of food establishments per 100,000 inhabitants) and the availability of food establishments (here defined as the entity of fast-food restaurants, supermarkets and other grocery stores) on zip code basis: 'ZIP Code Business Patterns' were utilized to assess economic activity of every registered establishment within zip code areas of Harris County.
Paquet et al., 2010	Density of fast-food restaurants 500m, (Food Environment, Density)	The proportion of restaurants located within 500-m (less than 6 minutes walking distance) of participants' residences that were classified as serving primarily fast food (i.e., number of fast-food restaurants/total number of restaurants).
Tsiampalis et al., 2021	Land covered by green urban spaces (by 10% increment), (Greenspace, Density)	Effect of 10% change of the municipalities' area covered by green urban spaces
Tsiampalis et al., 2021	Land covered by sports facilities (by 10% increment), (Greenspace, Density)	Effect of 10% change of the municipalities' area covered by sports facilities
Mancus et al., 2021	NDVI - Normalized Difference Vegetation Index, (Greenspace, Density)	Calculated normalized difference in vegetation index using the formula NDVI = (NIR-RED)/(NIR + RED) in 30m pixels for the City of Baltimore and took the average of the NDVI scores within 100m in a circular polygon (buffer) around participants' addresses.
Ribeiro et al., 2019	Green space at 400 m from school (yes), (Greenspace, Density)	Green space available within 400m of school
Ribeiro et al., 2019	Green space at 800 m from school (yes), (Greenspace, Density)	Green space available within 800m of school
Ribeiro et al., 2019	No. of green spaces at 400 m from school, (Greenspace, Density)	Number of green spaces available within 400m from the school (count)

Ribeiro et al., 2019	No. of green spaces at 800 m from school, (Greenspace, Density)	Number of green spaces available within 800m from the school (count)
Ribeiro et al., 2019	Distance to the nearest green space (km), (Greenspace, Distance)	Nearest greenspace measured in kilometresmeters
Ribeiro et al., 2019	Green space at 400 m from residence (yes), (Greenspace, Density)	Green space available within 400m of residential address
Ribeiro et al., 2019	Green space at 800 m from residence (yes), (Greenspace, Density)	Green space available within 800m of residential address
Ribeiro et al., 2019	No. of green spaces at 400 m from residence, (Greenspace, Density)	Number of green spaces available within 400m of residential address
Ribeiro et al., 2019	No. of green spaces at 800 m from residence, (Greenspace, Density)	Number of green spaces available within 800m of residential address
Ribeiro et al., 2019	Home garden (yes), (Greenspace, Density)	Garden available at residential address
Knobel et al., 2021	Perceived access to green space (% of adults reporting), (Greenspace, Density)	Used survey data to estimate the percent of all residents in a census tracts aged 18 years and older reporting perceived park or outdoor space access, based on responses to the question: "Is there a park or other outdoor space in your neighborhood that you are comfortable visiting during the day?".
Knobel et al., 2021	Percent tree canopy cover (%), (Greenspace, Density)	Used high-resolution (30.5 cm × 30.5 cm) orthophotography and Light Detection and Ranging (lidar) based land cover data to calculate percent tree canopy cover within each census tract. Percents were defined as the total area covered by tree canopy divided by the total land area in each census tract (km2). Estimates were derived by linearly interpolating values across assessments made in 2008 and 2018.
Knobel et al., 2021	Percent vegetation cover (%), (Greenspace, Density)	Used high-resolution (30.5 cm × 30.5 cm) orthophotography and Light Detection and Ranging (lidar) based land cover data to calculate percent vegetation cover within each census tract. Percents were defined as the total area covered by tree canopy plus grass/shrub cover divided by the total land area in each census tract (km2). Estimates were derived by linearly interpolating values across assessments made in 2008 and 2018.
Knobel et al., 2021	NDVI - Overall Greenness, (Greenspace, Density)	Created an annual mean NDVI score at the census tract level using images taken every 16 days in 2013 at a resolution of 250-metresmeters from the Moderate-resolution Imaging Spectroradiometer

		(MODIS) of NASA's Terra satellite (MOD13Q1, Version 6 product).
Walker et al., 2022	NDVI - Max, (Greenspace, Density)	95th NDVI percentile selected to represent the intensity of the greenest areas (P95) that may serve as local attractants or forested areas within a walking zone,
Walker et al., 2022	NDVI - Min, (Greenspace, Density)	Non-green areas (5th percentile NDVI) that are characteristic of a dense urban or industrial built environment (greyspace) and bare land, hypothesised to exhibit negative effects
Walker et al., 2022	NDVI - Standard Deviation, (Greenspace, Density)	Standard deviation in NDVI standard deviation represents variability in the amount and intensity of greenspace within each walking zone (measured in walking distance minutes)
Walker et al., 2022	NDVI - median, (Greenspace, Density)	Represents overall local greenspace levels
Sarkar et al., 2018	NDVI - Mean Quartiles, (Greenspace, Density)	An objective index of relative overall green vegetation or biomass derived from pixel values of spectral reflectance in remotely sensed data. This was modelled from a series of very high resolution (0.50 cm by 0.50 cm) BlueSky colour infrared (CIR) imagery derived from specially developed sensors mounted underneath a survey aircraft. Residential greenness acted as a proxy of salutogenic potential and was expressed as mean and standard deviation in the NDVI values within 500-m catchment radius of geocoded UK Biobank participants' dwellings.
Egorov et al., 2017	NDVI - LIDAR, (Greenspace, Density)	Used 1-metre resolution EnviroAtlas aerial photography data from the U.S. Department of Agriculture 2010 National Agriculture Imagery Program (NAIP). This data classifies land-cover into 5 categories: water; impervious surface; soil & barren; trees & forest, and; grass and other herbaceous. They considered total vegetated land cover as the proportion of land within either the 'trees & forest' or 'grass & other herbaceous' categorisations.

Walker et al., 2022	DRI-GLUCoSE Index, (Greenspace, Composite Measure)	Scaled so that high index values correspond with socially deprived areas and low greenspace. The diabetes risk index was calculated using principal component analysis (PCA) involving 11 SES (government transfer payments, lone parent families, household median income, prevalence of low income, unemployment rate, education-no degree, individual mean income, private dwellings-owned, private dwellings-rented, labour force participation rate, commute active) and 4 greenspace (NDVI - median, NDVI-5th percentile, NDVI - 95th percentile, NDVI - standard deviation) variables. The resulting loadings from the first component (explaining 49.3% of variance) were then applied as variable weights for the 15 input variables, with the sum rescaled to a range of − 1, 1 and taken as the participant DRI-glucose score.
Tsiampalis et al., 2021	No. of street markets/week (per 100,000 population; by 10 markets per 100,000 population increment), (Land-use, Density)	Effect of 0.0001% increase in number of street markets per 100,000 of the population
Tsiampalis et al., 2021	No. of supermarkets (per 100,000 population; by 10 markets per 100,000 population increment), (Land-use, Density)	Effect of 0.0001% increase in number of supermarkets per 100,000 of the population
Christine et al., 2015	Density of Commercial recreational establishments, (Land-use, Density)	Number of Commercial Recreational Establishments (gyms, pools, etc.) per square mile with densities mapped annually to reflect changes over time or if a participant moved.
Sarkar et al., 2018	Density of Retail, (Land-use, Density)	Measured as the number of retail outlets within 1-Km street catchment of a participant's geocoded dwelling and expressed as units/Km2.
Malambo et al., 2018	Distance to Community Centre, (Land-use, Distance)	Road distance buffer to the nearest community center (defined as a complex that included a police station, health clinic and open space), 500 m, 1000 m, or 1600 m from participant's household
Malambo et al., 2018	Distance to Shopping Centre, (Land-use, Distance)	Road distance buffer to the nearest shopping centre, 500m, 1000m, or 1600m from participant's household
Malambo et al., 2018	Distance to Taxi Rank, (Land-use, Distance)	Road distance buffer to the nearest taxi rank, 500m, 1000m, or 1600m from participant's household

Dengel et al., 2009	Distance to convenience/gas station network, (Land-use, Distance)	Distance to nearest convenience store or gas station was calculated by network and straight-line route. Network refers to a route between a participant's home and a specific feature that can be reached by someone on foot along a street network. A straight-line distance refers to the straight-line distance from the participant's home, regardless of street patterns.
Li et al., 2009	Walkability Index, (Land-use, Composite Density Measure)	Calculated on the basis of a composite score consisting of land use mix, street connectivity, number of public transit stations, and amount of green and open spaces.
Hamano et al., 2012	Road Network Distance, (Land-use, Distance)	Road distance from residence to 'population centre' (defined as the Shimane prefectural government; prefecture capital, Matsue city)
Hajna et al., 2018	Neighborhood active-living environment index, (Land-use, Composite Density Measure)	The z-scores of street connectivity (the number of ≥3-way intersections/km2 in each neighborhood), population density (census population counts/km2 in the dissemination area that corresponded to each postal code), and land use mix (representing the degree of heterogeneity in residential, commercial, institutional/governmental, and recreational land uses) were summed to create a neighborhood active-living environment index. A higher index represented neighborhoods with more potential to be active-living-friendly by having more connected street networks, greater intermixing of land uses, and more people.
de Courrèges, 2021	Walkability Index 2, (Land-use, Composite Density Measure)	Included measures within a 500-metre buffer of participant addresses. The included measures were: residential density (number of houses and apartments divided by available land area); street connectivity (the number of intersections with 3 or more walkable road segments within the buffer), and; land-use diversity (based on the number and types of destination reachable on foot within the area). The walkability index was calculated as the sum of the standardized z-scores of each indicator.
de Courrèges, 2021	The WS, (Land-use, Composite Density Measure)	Proprietary index which calculates walkability within a 1600-metre radial buffer based on: land-use diversity (with points based on distances to dining & drinking facilities, grocery shops, other shops, places for errands, parks, schools, and culture & entertainment); population density; block length, and; intersection density.
Chai et al., 2016	Tract urban area (vs. rural area), (Land-use, Density)	Defined using the U.S. Department of Agriculture (USDA) 2000 primary Rural-Urban Commuting Areas (RUCA) codes into ten detailed rural/urban categories. Urban defined as all metro tracts

		(RUCA=1–3, areas with a population of at least 50,000 people) and rural as all non-metro tracts (RUCA=4–10, areas with a population of less than 50,000 people)
Baldock et al., 2018	Distance to Public Open Spaces, (Land-use, Distance)	Road network distance measured from residential address to the nearest Public Open Space (POS) expressed as walking time (in minutes) based on a moderate adult walking speed of 4.8 km (3.0 miles) per hour. POS was defined as either (i) publicly owned land parcels larger than 700 m squared (m2)) or (ii) publicly accessible outdoor sporting facility/s and classified as active (e.g., sporting facilities), or passive (e.g., reserves). A single POS could include multiple land parcels; therefore, to avoid over-counting, parcels within a five-metre adjacency were merged to create a single land parcel.
Baldock et al., 2018	Discordance between perceived and objective measures of distance to Public Open Space, (Land-use, Distance)	Discordance between Perceived and Objective Distances (Overestimation of Distances) to Fruit and Vegetable Retailers and Public Open Space Overestimation of the actual distance to FVR and POS represented discordance between perceived and objective distances. The difference between perceived and objective distance scores was calculated as objective less perceived distance.
Baumgardner et al., 2006	Driving Distance to Clinic, (Land-use, Distance)	Calculated driving (road) distance between residential address and 2 types of healthcare clinic. Distances to clinics were categorized as short (1-mile), medium (2-miles), or long (3-miles) for each clinic by observation of natural break points on histograms of the distribution of the distances to two clinics.
Christine et al., 2015	Physical Activity Summary Score, (Land-use, Composite Density Measure)	A composite score of 1) No. Of commercial recreational establishments (gyms, pools, etc) per square mile, with densities mapped annually to reflect changes over time/address changes &; 2) survey of perceived availability of opportunities to be physically active within the neighborhood (Likert scale 1-5, higher values represent more favourable environment).
Walker et al., 2022	Urbanity level, (Land-use, Density)	Neighborhood type, categorised as either suburban/rural or urban
Sarkar et al., 2018	Public Transport Density, (Land-use, Density)	Measured as the number of public transport stops within 1-Km street catchment of a participant's geocoded dwelling and expressed as units/Km2.
Sarkar et al., 2018	Slope variability, (Land-use, Density)	Mean terrain (deg.) Was used as a measure of terrain. The effect of terrain variability was operationalized with the help of a 5 m resolution BlueSky digital terrain model, expressed in terms of variability (standard deviation) in slope in degrees within a 500-m catchment of dwelling

Sarkar et al., 2018	Street movement density, (Land-use, Density)	Street-level movement density was modelled from the underlying topology of street network design as the simulated counts of movement through each link in the network, given its relative position and the connectivity with other links within the network. Network analyses were performed on the Ordnance Survey Integrated Transport Network database comprising 5 million street links within a 50 Km radius of each of the UK Biobank assessment centres (Cooper et al., 2012). Movement density was measured from the underlying street configuration, expressed in terms of the graphical metrics of betweenness centrality or through-movement potential within the defined street catchment of UKB participants' dwelling and acted as proxy of walking density (Sarkar et al., 2015b),
Sarkar et al., 2018	Destination accessibility (mean street network distance), (Land-use, Distance)	Measures street network proximity to behaviourally-relevant nearest destinations (schools, medical facilities, leisure, retail and places of worship within the defined network catchment) measured in metresmeters from the geocoded dwelling location in ArcGIS Network Analyst and a mean index of destination accessibility was developed for each study participant.
Sarkar et al., 2018	Residential Density, (Land-use, Density)	Measured as the number of dwelling units (including detached, semi-detached, terraced and self-contained flats), within 1-Km street catchment of a participant's geocoded dwelling and expressed as units/Km2.
Sarkar et al., 2018	Walkability Index - Quartiles, (Land-use, Composite Density Measure)	Considered an overall neighborhood walkability index within a 1-Km street catchment. This included: density of housing; density of retail; density of public transport street-level movement density (a summary score of network distance and connectedness), and; network-proximity (to schools, medical facilities, leisure, retail and places of worship within the defined network catchment). Each of these components was ranked into deciles, with destination distance being reversed so that further distances represented poorer walkability scores, creating a score of 5-50, with higher scores representing greater walkability.
